# Different post-mortem brain regions from three Chinese FFI patients induce different reactive profiles both in the first and second generation RT-QuIC assays

**DOI:** 10.1080/19336896.2020.1782809

**Published:** 2020-06-23

**Authors:** Kang Xiao, Qi Shi, Wei Zhou, Xiao-Ping Dong

**Affiliations:** a State Key Laboratory for Infectious Disease Prevention and Control, Collaborative Innovation Center for Diagnosis and Treatment of Infectious Diseases (Zhejiang University), National Institute for Viral Disease Control and Prevention, Chinese Center for Disease Control and Prevention, Beijing, China; b Center for Global Public Health, Chinese Center for Disease Control and Prevention, Beijing, China; c Wuhan Institute of Virology, Chinese Academy of Science, Wuhan, China; d China Academy of Chinese Medical Sciences, Beijing, China

**Keywords:** Prion, FFI, RT-QuIC, brain region, PK-resistance

## Abstract

Fatal Familial Insomnia (FFI) is one of the most popular genetic prion disease (gPrD) in China. Unlike the other types of human prion diseases, FFI patients show distinctive neuropathological characteristics, such as less deposition of PrP^Sc^, low tissue infectivity and severe neuron losses in some special brain regions. Compared with other gPrDs, the positive reactions of cerebrospinal fluid (CSF) RT-QuIC of FFI patients were markedly low. However, the reactivities of RT-QuIC of the brain tissues, particularly different brain regions, of FFI cases are rarely described. In this study, three different brain regions from three FFI patients were subjected into two kinds of RT-QuIC assays using recombinant hamster PrP23-231 (rHaPrP23-231) and PrP90-231 (rHaPrP90-231) as the substrates, respectively. The results showed that the general RT-QuIC reactivities of the brains from FFI cases were significantly lower than that of sCJD. Analyses of the positive rates and the reactivities (lag time and rfu peak) of RT-QuIC identified that the homogenates of frontal lobe induced the most active reaction, followed by thalamus and callosum. The RT-QuIC reactivity in the tested brain sample was closely associated with the intensity of PK-resistant PrP^Sc^. Moreover, we also verified that the sensitivity of the RT-QuIC of rHaPrP90-231 was much higher than that of rHaPrP23-231. Those data confirm that brain tissues of FFI patients are able to convert positive reactions in RT-QuIC and show regional-associated positive converting capacities.

## Introduction

Fatal familial insomnia (FFI) is a special kind of inherited human prion disease with distinct clinical and neuropathological features compared with other human prion diseases, such as sporadic Creutzfeldt-Jacob disease (sCJD), Gerstmann-Sträussler-Scheinker diseases (GSS) [1–3]. A special mutation from aspartic acid to asparagines at the position of aa 178 (D178 N) in *PRNP* is directly linked with the onset of FFI. Unlike sCJD and other genetic Creutzfeldt-Jakob disease (gCJD), spongiform degeneration and PrP^Sc^ deposits are not frequent in the brains of FFI, instead of severe neuron loss and active astrogliosis in some brain regions, especially thalamus [4].

FFI cases with D178 N mutation have been reported in different races of many countries and regions. Based on the surveillance data for human prion disease in China, D178 N FFI is most frequently detected genetic prion disease [5]. Previously, we have thoroughly described the epidemiological, clinical, genetic and laboratory characteristics of 40 Chinese D178 N FFI patients [5]. We have reported that 16.7% Chinese FFI cases show positive in RT-QuIC assays of cerebrospinal fluid (CSF), using recombinant hamster PrP from aa 23 to 231 (rHaPrP23-231) as the substrate, which is remarkably lower than T188 K gCJD that is the second common gCJD and E200 K gCJD that is the third common gCJD [6].

Compared with sCJD, the infectivity of the tissues of central nerve system (CNS) of FFI patients on the susceptible experimental animals is extremely low. It may link with the less amount of PrP^Sc^ in brain tissues of FFI. It may also reflect the different conformational structure of FFI prion. In this study, with the post-mortem brain tissues of three Chinese FFI cases, we tested the reactivities of three different brain regions in two kinds of RT-QuIC assays, using rHaPrP23-231 or recombinant hamster PrP from aa 90 to 231 (rHaPrP90-231) as the substrate. We found that the brain homogenates of FFI cases induced positive conversion in RT-QuIC. The positive converting ability of FFI brains was lower than that of sCJD brains. The lysates of frontal lobes of FFI and sCJD patients possessed apparently stronger converting abilities than that of thalamus and callosum. In addition, RT-QuIC assay of rHaPrP90-231 was markedly more sensitive than that of rHaPrP23-231.

## Materials and methods

### Ethics statement

The present study was approved by the Ethical Committee of the National Institute for Viral Disease Control and Prevention (Beijing, China) under the protocol 2009ZX10004-101, including the usages of the storage brain samples of FFI and sCJD patients from Chinese CJD surveillance network.

### Expression and purification of the hamster recombinant PrP (rPrP) protein

Recombinant hamster PrP proteins (residues 23–231 and 90–231; GenBank accession no. K02234) were prepared according to the method described previously [6,7]. Briefly, the two DNA sequences of hamster PrP residues 23–231 and 90–231 were firstly ligated into the pRSET vector (cat. no. V35120; Thermo Fisher Scientific, Inc., Waltham, MA, USA), and then the recombinant plasmids were transformed into BL21(DE3)pLysS competent cells (cat. no. C1500; Beijing Solarbio Science & Technology Co., Ltd., Beijing, China). After treated the bacteria pellets by ultrasonication, the lysates were denatured with guanidine-HCL and the expressed proteins were purified by chromatography using Ni-NTA Superflow resin (cat. no. 30,430; Qiagen, Hilden, Germany) in an XK 16/40 column (GE Healthcare Life Sciences, Little Chalfont, UK) at a flow rate of 2.3 ml per min. The purified proteins were dialysed into 10 mM sodium phosphate buffer (pH 5.8) and the concentrations of recombinant PrP proteins (rHaPrP23-231 and rHaPrP90-231) were adjusted to 500 µg/ml as determined by absorbance measured at 280 nm. Following filtration (0.22 µm syringe filter, cat. no. SLGP033RB, Merck millipore), the recombinant PrP proteins were aliquoted and stored at −80°C, respectively.

### Brain samples of FFI patients

The storage brain samples from three Chinese FFI patients were enrolled in this study, including the brain regions of frontal lobe, thalamus and callosum. The exact epidemiological, clinical, genetic and neuropathological data of these three cases were well documented previously [8].

### Preparation of brain homogenate (BH)

Ten per cent BHs of FFI and sCJD patients were prepared in lysis buffer (100 mM NaCl, 10 mM EDTA, 0.5% Nonidet P-40, 0.5% sodium deoxycholate, 10 mM Tris, pH7.5) according to a previously described protocol [9,10]. To set up the positive and negative controls for RT-QuIC, 10% BHs of the hamsters infected with scrapie agent 263 K and normal hamsters were prepared as well. BH preparation of 10^-5^ dilution of each specimen was used as the seed for RT-QuIC.

### PK-resistant assay

To detect the PK resistances of PrP in the brains of FFI patients, the BHs were exposed to different amounts of PK at the final concentrations of 5, 10, 15, 20 and 25 μg/ml at 37°C for 60 min prion to PrP-specific Western blot. Briefly, after separating in 12% SDS-PAGE, the proteins were transferred onto a nitrocellulose filter membrane (cat. no. 10,600,001; GE Healthcare Life Sciences). The membranes were incubated with 1:1000 diluted commercial PrP-specific monoclonal antibody (mAb) 6D11 (cat. no. 58,581; Santa) at 4°C overnight, and subsequently, with a horseradish peroxidase-conjugated goat anti-mouse IgG secondary antibody (cat. no.31430; Thermo Fisher Scientific, Inc.) at 37°C for 2 h with a dilution of 1:5,000.

### RT-QuIC assay

The details of the RT-QuIC assay was described previously [6]. Briefly, RT-QuIC reaction contained 1 µl 10^−5^ diluted BH, 1X PBS, 170 mM NaCl, 1 mM EDTA, 0.01 mM ThT, 0.001% SDS and 10 µg of rHaPrP23-231 or rHaPrP90-231 in a final volume of 100 µl. Each sample was assayed in quadruplicated. The assay was conducted in a black 96-well, optical-bottomed plate (Nunc, 265,301) on a BMG FLUOstar plate reader (BMG LABTECH). The main working conditions were fixed as follows: temperature, 50°C; vibration speed, 900 rpm; vibration/incubation time, 90/30 sec; total reaction time, 90 h. ThT fluorescence (excitation wavelength, 450 nm; emission wavelength, 480 nm) each reaction was automatically measured every 30 min and expressed as relative fluorescence units (rfu). The cut-off value was set as the mean value of the negative controls plus 3 times the standard deviation. A sample was considered to be positive when ≥2 wells revealed positive reaction curves.

### Statistical analysis

Quantitative analyses of immunoblot images were carried out using software Image J. Data shown were means ± SD of triplicate samples from a single experiment and were representative of three independent experiments. Statistical analyses were performed using Student’s t-test.

## Results

**Figure 1.**
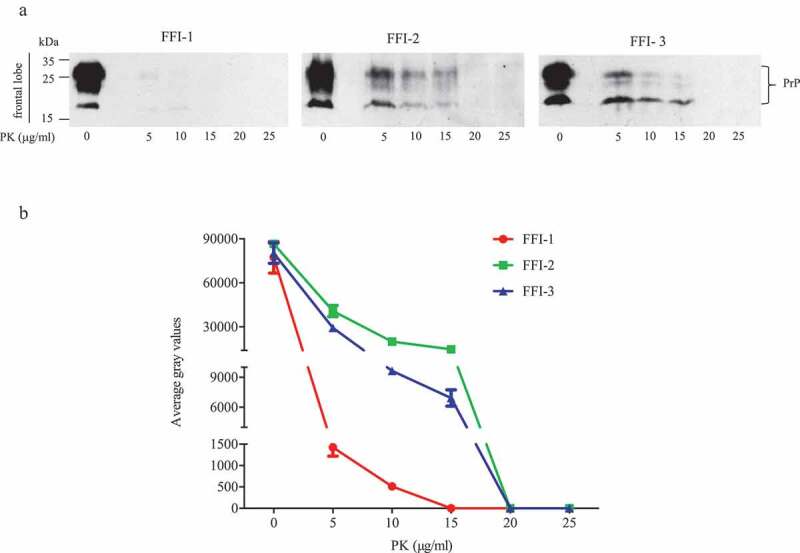
PK-resistances of PrP^Sc^ in the frontal lobe of 3 FFI patients. (a). Western blot of BH digested by 0, 5, 10, 15, 20 and 25 μg/ml of PK at 37°C for 60 min. (b). Quantitative analyses of each grey numerical value of PrP blots. Data shown were means ± SD of triplicate samples. Student’s t-test was used for statistical analyses.

To access the reactivities of the brains of FFI patients in the RT-QuIC assays, the homogenates of thalamus, callosum and frontal lobe of three Chinese FFI patients were prepared. The epidemiological, clinical, genetic and neuropathological data were thoroughly described previously [8]. To verify the PK-resistances of PrP^Sc^ in the prepared brain homogenates again, different concentrated PK from 5 to 25 μg/ml were introduced into the lysates of frontal lobe prior to Western blot. As shown in Figure 1(a), very weak PrP bands were observed in the preparations containing 5 and 10 μg/ml PK of FFI-1 case. The PK-resistances of PrP in the preparations FFI-2 and FFI-3 were more apparent, revealing marked PK-resistant signals in the reactions containing 5, 10 and 15 μg/ml PK. PK-resistant signals were undetectable in all preparations containing 20 and 25 μg/ml PK. Quantitative assays of the average grey values of PK-resistant PrP signals showed dose-dependent decreases along with the increase of PK amounts (Figure 1(b)).

**Figure 2.**
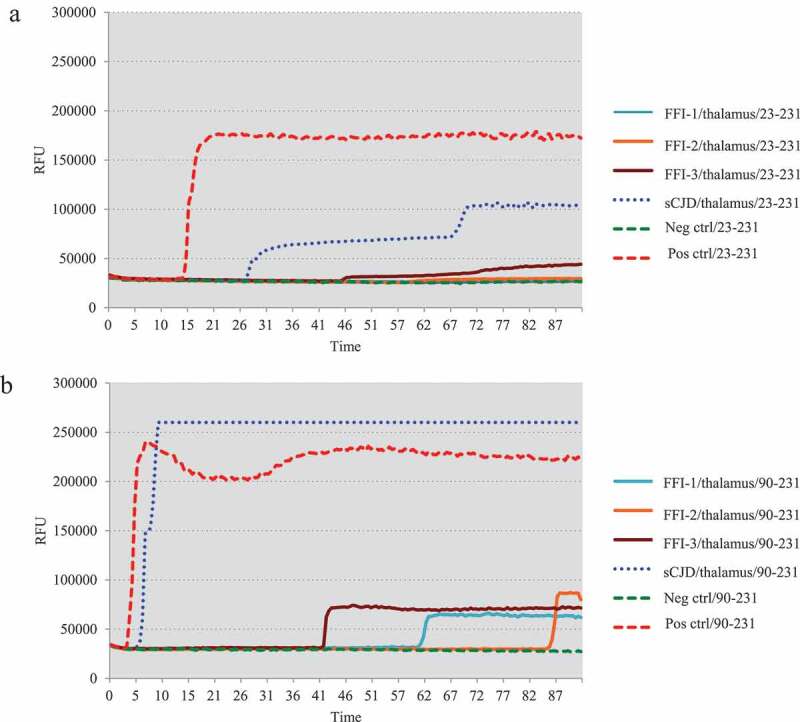
Results of RT-QuIC assay for detecting PrPSc in thalamus of 3 FFI patients with two different substrate. BHs including FFIs and controls were all 10^−5^ diluted. Normal and sCJD BHs were used as negative and positive control, respectively. (a). The substrate of RT-QuIC was hamster rPrP23-231. (b). The substrate of RT-QuIC was hamster rPrP90-231.

**Figure 3.**
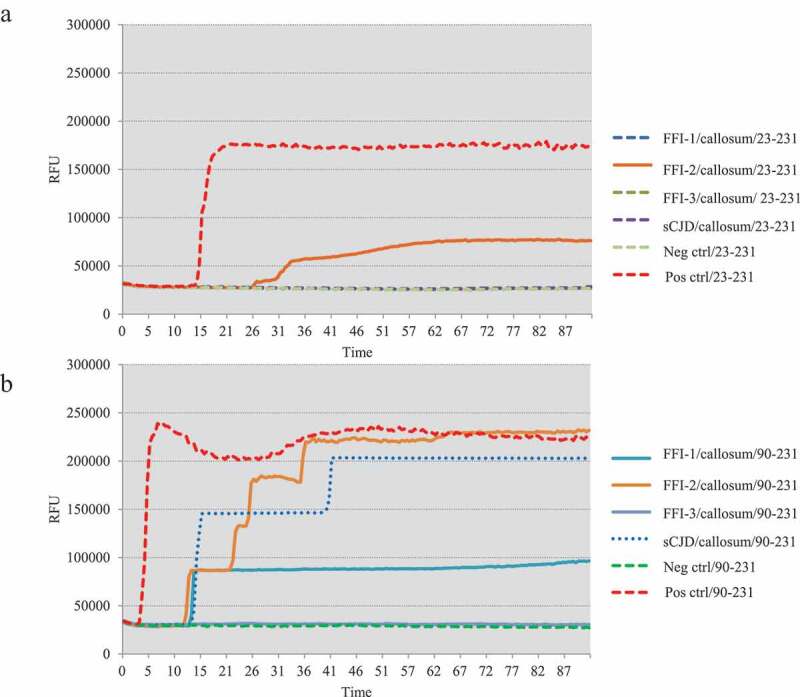
Results of RT-QuIC assay for detecting PrPSc in callosum of 3 FFI patients with two different substrate. BHs including FFIs and controls were all 10^−5^ diluted. Normal and sCJD BHs were used as negative and positive control, respectively. (a). The substrate of RT-QuIC was hamster rPrP23-231. (b). The substrate of RT-QuIC was hamster rPrP90-231.

**Figure 4.**
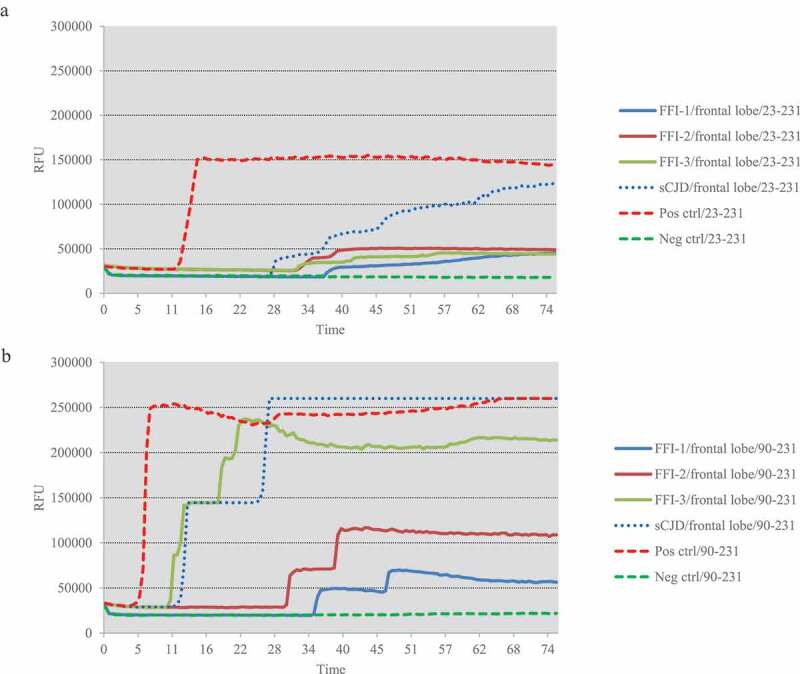
Results of RT-QuIC assay for detecting PrPSc in frontal lobe of 3 FFI patients with two different substrate. BHs including FFIs and controls were all 10^−5^ diluted. Normal and sCJD BHs were used as negative and positive control, respectively. (a). The substrate of RT-QuIC was hamster rPrP23-231. (b). The substrate of RT-QuIC was hamster rPrP90-231.

One microlitre of 10^−5^ diluted various brain homogenates were subjected into the RT-QuIC assays using rHaPrP23-231 and rHaPrP90-231 as the substrates, respectively, together with 10^−5^ diluted cortical homogenate of scrapie agent 263 K-infected hamster as the positive control for RT-QuIC. Meanwhile, same amounts of the brain lysates from an sCJD case were also administrated as the control. PK digested PrP-specific Western blots verified the presences of PrP^Sc^ in the homogenates of thalamus, callosum and frontal lobe. In the tests of thalamus, no positive reactions were observed in all three cases in the RT-QuIC of rHaPrP23-231, whereas a clear positive reaction was recorded in the test of sCJD (Figure 2(a)). A slight increase of reactive curve was recorded in the test of FFI-3, but its rfu value was lower than two times of negative control (Figure 2(a)). In the RT-QuIC of rHaPrP90-231, the thalamus lysate of sCJD induced remarkable positive reaction with short lag time and high rfu value, while all three FFI preparations also showed positive converting curves despite lower rfu values at late stages of reactions (Figure 2(b)). In the tests of callosum, only FFI-2 showed positive in the RT-QuIC of rHa23-231, while FFI-1, FFI-3 and sCJD were all negative (Figure 3(a)). In the RT-QuIC of rHaPrP90-231, sCJD and FFI-1 converted positive but with relatively long lag times and low rfu values compared with FFI-2, whereas FFI-3 was still negative (Figure 3(b)). In the test of frontal lobe, all three FFI preparations and sCJD case converted to positive both in the RT-QuIC of rHaPrP23-231 and rHaPrP90-231 (Figure 4(a,b)). In line with the tests of other two brain regions above, the reactivities of all preparations in the RT-QuIC of rHaPrP90-231 were obviously stronger than those of rHaPrP23-231.

The reactivities of three brain homogenates from three FFI cases and one sCJD case in two different RT-QuIC techniques are summarized in Table 1, including the lag times and peak of rfu values. Obviously, the brain lysates of FFI cases were able to induce positive reactions in RT-QuIC, in which FFI-2 and FFI-3 cases showed stronger converting abilities than FFI-1. The reactivity of the brains of the sCJD patient in RT-QuIC was stronger than those of FFI cases. The lysates of frontal lobes of FFI and sCJD patients possessed markedly stronger converting abilities than that of thalamus and callosum. Additionally, the RT-QuIC assay of rHaPrP90-231 showed markedly higher sensitivity than that of rHaPrP23-231.

**Table 1.** The RT-QuIC reactivities of the lysates of three different brain regions from three FFI cases.

Substrate	Brain region	Case	Lag time (hr)	Peak of rfu
PrP23-231	Thalamus	FFI-1	NA	NA
		FFI-2	NA	NA
		FFI-3	NA	NA
		sCJD	29	105,000
	Callosum	FFI-1	NA	NA
		FFI-2	32	77,000
		FFI-3	NA	NA
		sCJD	NA	NA
	Frontal lobe	FFI-1	37	45,000
		FFI-2	33	50,000
		FFI-3	34	45,000
		sCJD	28	71,000
PrP90-231	Thalamus	FFI-1	62	65,000
		FFI-2	87	86,000
		FFI-3	42	74,000
		sCJD	6	260,000
	Callosum	FFI-1	14	88,000
		FFI-2	13	230,000
		FFI-3	NA	NA
		sCJD	14	200,000
	Frontal lobe	FFI-1	35	69,000
		FFI-2	30	110,000
		FFI-3	10	215,000
		sCJD	12	260,000

## Discussion

In this report, we have confirmed again that the brain tissues of FFI cases possess the apparent ability to induce the fibrillation in RT-QuIC. In line with the observations of other previous studies, the brain tissues from three tested Chinese FFI cases seem to be less capable to induce positive reactivity in RT-QuIC tests compared with those from sCJD cases at the same working dilution, although we do not titre the exact EC_50_ of the tested specimens [11]. Our recent article has also identified that the RT-QuIC reactivity of the cerebrospinal fluids (CSF) of FFI cases is markedly weaker than those of E200 K gCJD and T188 K gCJD patients [6]. Such phenomenon is considered to be understandable, since the amounts of PrP^Sc^ plaques in the brains of FFI cases are usually remarkably less than that of the patients of sCJD and other types of genetic prion diseases, such as E200 K gCJD [12–15]. In addition, the electrophoretic pattern of PrP^Sc^, particularly the glycosylation profile, in the brains of FFI cases is different from that of most sCJD cases, revealing predominate-diglycosylated PrP^Sc^ [12,15]. It has been proposed that the reactivities in RT-QuIC of human prion diseases may differ largely, not only due to the amounts of PrP^Sc^ but also related with the subtypes of PrP^Sc^ in the brain tissues [16]. The CSF specimens from the sCJD patients with the type of MM1 PrP^Sc^ show stronger reactivity in RT-QuIC than those with the type of MM2 PrP^Sc^ [11]. It is reasonable to assume that, besides less PrP^Sc^ amount, the unknown-refined conformational diversity of PrP^Sc^ in the brains of FFI cases might also contribute to the low reactivity in the RT-QuIC assays.

Our data here also illustrate that the RT-QuIC reactivities of the different brain regions of FFI cases vary apparently, the frontal lobe with strongest RT-QuIC reactivity and the callosum with the weakest one. Such region-associated appearance has been observed not only in every individual case but also in the two different RT-QuIC assays. The region-dependent reactivity in RT-QuIC is also described in other studies [17]. The cerebral cortexes of FFI patients are more likely to induce positive RT-QuIC results, while in the thalamus and cerebellum, the rates of positive results are significantly low [17]. We believe that this diversity associates with the difference in PrP^Sc^ amounts among various brain regions. Our previous neurological study on these three FFI cases has already showed relatively more PK-resistant PrP^Sc^ in the region of frontal lobe and remarkable less in callosum [8]. The close association between RT-QuIC reactivity and PrP^Sc^ amount is also observable in the tests of the individual cases. The FFI-1 patient in this study containing very weak PK-resistant PrP signal in the frontal lobe in Western blot shows long lag time and low rfu value in RT-QuIC assays.

It is not surprising that RT-QuIC technique using rHaPrP90-231 as the substrate shows markedly higher sensitivity in detecting the prions in the brain tissues of FFI and sCJD cases than that using rHaPrP23-231. As the second generation technique, RT-QuIC of rHaPrP90-231 has already shown reliable sensitivity and specificity in CSF RT-QuIC assays for sCJD [16]. Many CSF samples of sCJD showing negative in RT-QuIC of rHaPrP23-231 convert positive in the RT-QuIC assays of rHaPrP90-231 substrates, illustrating great feasibility in the diagnosis of sCJD using CSF and other peripheral tissues, such as skin specimen [18]. On the other hand, RT-QuIC supplies a sensitive and efficient tool for the study of prion biology and pathogenesis of prion diseases [19]. Definitely, RT-QuIC, especially the second generation RT-QuIC, will be a helpful technique to address the distribution and accumulation of abnormal prion proteins in different brain regions, as well as in peripheral organs and tissues, in prion diseases, particularly in FFI that usually contains less amount of PrP^Sc^.
